# More future synergies and less trade‐offs between forest ecosystem services with natural climate solutions instead of bioeconomy solutions

**DOI:** 10.1111/gcb.16364

**Published:** 2022-08-10

**Authors:** Adriano Mazziotta, Johanna Lundström, Nicklas Forsell, Helen Moor, Jeannette Eggers, Narayanan Subramanian, Núria Aquilué, Alejandra Morán‐Ordóñez, Lluís Brotons, Tord Snäll

**Affiliations:** ^1^ Swedish Species Information Centre Swedish University of Agricultural Sciences (SLU) Uppsala Sweden; ^2^ Natural Resources Institute Finland (Luke) Helsinki Finland; ^3^ Department of Forest Resource Management Swedish University of Agricultural Sciences (SLU) Umeå Sweden; ^4^ International Institute for Applied Systems Analysis (IIASA) Laxenburg Austria; ^5^ Swiss Federal Research Institute WSL Birmensdorf Switzerland; ^6^ Swedish University of Agricultural Sciences Southern Swedish Forest Research Centre Alnarp Sweden; ^7^ Forest Science and Technology Centre of Catalonia (CTFC) Solsona Spain; ^8^ Centre d' Étude de la Forêt (CEF) Université du Québec à Montréal (UQAM) Montréal Quebec Canada; ^9^ Centre for Research on Ecology and Forestry Applications (CREAF) Cerdanyola del Valles Spain; ^10^ CSIC Cerdanyola del Valles Spain

**Keywords:** bioeconomy, bioenergy, boreal forest, climate change, ecosystem services, EU biodiversity strategy, EU forest strategy, GLOBIOM, natural climate solutions

## Abstract

To reach the Paris Agreement, societies need to increase the global terrestrial carbon sink. There are many climate change mitigation solutions (CCMS) for forests, including increasing bioenergy, bioeconomy, and protection. Bioenergy and bioeconomy solutions use climate‐smart, intensive management to generate high quantities of bioenergy and bioproducts. Protection of (semi‐)natural forests is a major component of “natural climate solution” (NCS) since forests store carbon in standing biomass and soil. Furthermore, protected forests provide more habitat for biodiversity and non‐wood ecosystem services (ES). We investigated the impacts of different CCMS and climate scenarios, jointly or in isolation, on future wood ES, non‐wood ES, and regulating ES for a major wood provider for the international market. Specifically, we projected future ES given by three CCMS scenarios for Sweden 2020–2100. In the long term, fulfilling the increasing wood demand through bioenergy and bioeconomy solutions will decrease ES multifunctionality, but the increased stand age and wood stocks induced by rising greenhouse gas (GHG) concentrations will partially offset these negative effects. Adopting bioenergy and bioeconomy solutions will have a greater negative impact on ES supply than adopting NCS. Bioenergy or bioeconomy solutions, as well as increasing GHG emissions, will reduce synergies and increase trade‐offs in ES. NCS, by contrast, increases the supply of multiple ES in synergy, even transforming current ES trade‐offs into future synergies. Moreover, NCS can be considered an adaptation measure to offset negative climate change effects on the future supplies of non‐wood ES. In boreal countries around the world, forestry strategies that integrate NCS more deeply are crucial to ensure a synergistic supply of multiple ES.

## Graphical abstract

We investigated future impacts of climate change mitigation solutions and climate scenarios on future ecosystem services (ES) for Sweden, a major provider of wood for the international market. We found that bioenergy‐ or bioeconomy‐focused solutions increasing wood demand will decrease future ES supply and ES multifunctionality. It will also decrease ES synergies and instead increase future ES trade‐offs. In contrast, natural climate solutions increasing forest protection will increase future ES synergies, transform current ES trade‐offs into future ES synergies and offset the negative effects of increasing greenhouse gas concentrations on non‐wood ES.

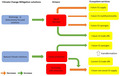

## 1 INTRODUCTION

**FIGURE 1:**
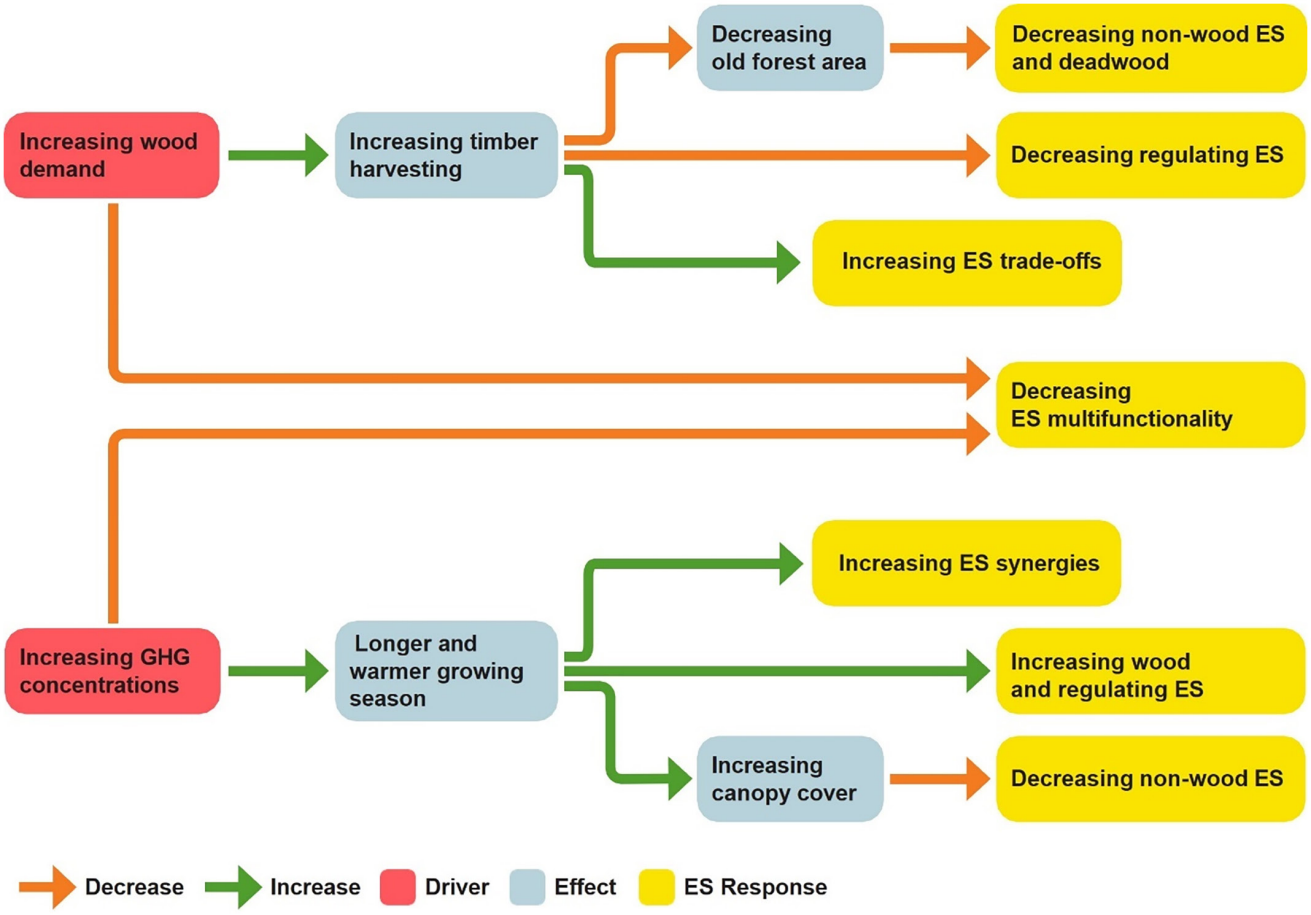
Expectations on how increasing wood demand and greenhouse gas (GHG) concentrations will change forest management and forest structure, thereby changing ecosystem services (ES) supply and multifunctionality of the northern European forest.

To stabilize global warming well under 2°C above pre‐industrial levels by 2100 (the Paris Agreement), societies act to increase the terrestrial carbon sink. In forests, bioenergy and bioeconomy solutions, and natural climate solutions (NCS) are adopted (Creutzig et al., 2015; Griscom et al., 2017; Shukla et al., 2019; Wu et al., 2020). NCS, that is, protection by setting‐aside (semi‐)natural forests means storing carbon in standing biomass and soil (Alrahahleh et al., 2017; Heinonen et al., 2017; Pohjanmies et al., 2021). Bioenergy or bioeconomy solutions are instead implemented at large scales by managing forest to increase the wood harvesting (Heinonen et al., 2018) to produce bioenergy or bioproducts, respectively (Persson & Egnell, 2018; Soimakallio et al., 2016; Vanhala et al., 2013). However, an increased wood demand with bioenergy and bioeconomy solutions, linked to population growth, globalization and climate change mitigation (Jonsson, 2013), reduces the climate change mitigation capacity of standing forests (Repo et al., 2015) and their soils (Achat et al., 2015; “regulating ecosystem services [ES],” Figure 1; Table 1).

**TABLE 1:** List of the seven investigated ecosystem services (ES) and their categorization following Common International Classification of Ecosystem Services (CICES; Haines‐Young & Potschin, 2018) and Nature's Contributions to People (NCP; Díaz et al., 2018). For each service, we detail the related ecosystem functions as well as the ES bundle into which we group them in the current study

ES	ES category		Ecosystem function	ES bundle
	CICES	NCP		
Net wood biomass accumulation	Provisioning service	Regulation of climate	Primary production of biomass	Wood ES
	Regulating and maintenance services	Energy	C sequestration	
		Materials, companionship and labor		
		Supporting identities		
Tree C storage	Regulating and maintenance services	Regulation of climate	C sequestration	Regulating ES
Soil C storage	Regulating and maintenance services	Regulation of climate	C sequestration	Regulating ES
Deadwood volume	Regulating and maintenance services	Regulation of climate	Habitat provisioning	Wood ES
		Habitat creation and maintenance	C sequestration	
Bilberry production	Provisioning service	Food and feed	Primary production of berries	Non‐wood ES
	Recreational service	Physical and psychological experiences		
	Cultural service	Supporting identities		
Wildfood plants for game	Provisioning service	Food and feed	Secondary production	Non‐wood ES
	Recreational service	Physical and psychological experiences		
	Cultural service	Supporting identities		
Understory plant richness	Regulating and maintenance services	Habitat creation and maintenance	Habitat heterogeneity	Non‐wood ES
	Cultural service	Learning and inspiration		

Natural climate solution, bioenergy or bioeconomy solutions can either increase or decrease the supply of forest ES and biodiversity. Forests that are protected as part of NCS provide more habitat suitable for biodiversity and non‐wood ES compared to forests that are managed for bioenergy or bioeconomy solutions (Alrahahleh et al., 2017; Heinonen et al., 2017), which may even cause forest degradation if the forestry is too intensive (Selva et al., 2020). In Fennoscandian boreal forest, bioenergy or bioeconomy solutions are frequently applied and therefore, forests are often harvested when optimal from an economic point of view before they reach biological maturity (Esseen et al., 1997). These solutions may therefore retain less mature forest than NCS, and hence less habitat for understory plants supplying “non‐wood ES” like berries or wildfood plants for game (Table 1). Bioenergy and bioeconomy solutions also retain less old trees and consequently less deadwood habitat for biodiversity than NCS with protected forest (Figure 1; Heinonen et al., 2017; Jonsson et al., 2020). For these reasons, the solutions are also expected to supply lower ES multifunctionality than NCS (Jonsson et al., 2020; Figure 1).

The impact of what we hereafter refer to as three climate change mitigation solutions (“CCMS”; bioenergy, bioeconomy, and NCS) on ES is modulated by climate change (Morán‐Ordóñez et al., 2020). In the boreal zone, the increase in atmospheric greenhouse gas (GHG) concentrations, inducing a longer and warmer growing season (Subramanian et al., 2019), may enhance the supply of “wood ES”, while instead limiting the supply of other non‐wood ES (Table 1; Figure 1). Specifically, the increase in GHG concentrations and temperatures is likely to enhance biomass production (Poudel et al., 2011; Subramanian et al., 2019) and accumulation of carbon in trees and soil (Poudel et al., 2011) if the availability of nitrogen and water in the soil is not a limiting factor (Stinziano & Way, 2014). This may further increase the production of deadwood constituting habitat for biodiversity (Blattert et al., 2020), with deadwood amounts ultimately also depending on the management. Finally, increasing GHG concentrations may decrease the supply of non‐wood ES (Strengbom et al., 2018) by potentially making canopies denser (Hedwall et al., 2019; Figure 1). Specifically, more shady conditions are unfavorable for understory plants and undermine the production of berries and food‐source plants for game. Because of the decrease of these light‐demanding plant species, whose diversity delivers several ES, increasing GHG concentrations are expected to decrease forest ES multifunctionality (Figure 1).

Climate change and bioenergy and bioeconomy solutions are expected to have a contrasting effect on the local synergies versus trade‐offs between ES supply, that is, on the capacity of the forest to simultaneously supply multiple ES. Increasing GHG concentrations are indeed expected to increase ES synergies because of the potentially increased forest growth (Morán‐Ordóñez et al., 2020), but the increase in wood demand given by these solutions is expected to increase ES trade‐offs if forests are only managed for maximizing timber harvesting (Alcamo et al., 2005; Díaz‐Yáñez et al., 2021; Figure 1).

The supply of forest ES is thus affected by the alternative CCMS, but they also affect biodiversity preservation. Bioenergy and bioeconomy solutions promote mainly biomass production and carbon sequestration (cf. Baker et al., 2019; Kauppi et al., 2020; Makkonen et al., 2015), while NCS promote mainly biodiversity and non‐wood ES. However, integration of these CCMS has been described as pivotal to decrease the conflicts in the use of forest for timber, climate change mitigation, and biodiversity (Blattert et al., 2022; Elomina & Pülzl, 2021; Köhl et al., 2021). For example, in the EU, bioenergy and bioeconomy solutions have been described as curbing anthropogenic GHG concentration in the EU Renewable Energy Directive (European Commission, 2021a), and in the Land‐use, Land‐Use Change and Forestry Regulation and international climate targets (Shoeibi et al., 2015), but they do not consider the consequences for biodiversity. In contrast, the EU Biodiversity Strategy for 2030 (European Commission, 2021b) and the New EU Forest Strategy for 2030 (European Commission, 2021c) seek to maintain and restore forest ES also through NCS. To support the capacity of the forest to mitigate climate change, supply multiple ES and retain biodiversity, the development of forest strategies should implement CCMS that also meet biodiversity targets (Crossman et al., 2013). The possibility for cost‐efficient biodiversity preservation in forest strategies (Graham et al., 2021) will be better achieved through an understanding of the potential effects of CCMS on both future biodiversity and ES supply (Blattert et al., 2020). The reason is that certain CCMS involve an increase in timber harvesting (Baul et al., 2017; Gustavsson et al., 2017), and this may escalate conflicts in the use of the forest for both climate change mitigation and biodiversity preservation (Camia et al., 2021; Snäll et al., 2017).

We provide a first integrated analysis of the joint impacts of alternative CCMS and of increasing GHG concentrations on future wood, non‐wood ES, and biodiversity for a country that is a major wood producer for the international market. This includes highlighting potential incompatibilities between CCMS on future country‐scale ES and biodiversity (Lundmark et al., 2014; Nordström et al., 2016). Specifically, we simulate three scenarios of CCMS (*Current Policy*, *Bioenergy*, and *Bioeconomy*), representing a gradient of increasing timber harvesting to fulfill the increasing demand of wood given by these CCMS. *Bioeconomy* assumes extensive development of wood products not yet available on the market, in addition to the increased use of *Bioenergy* compared to *Current Policy*. All scenarios further include a fourth *Set‐aside* scenario, an example of NCS where the mitigation is achieved by storing carbon in standing biomass and soil. The success of CCMS depends on how the future climate develops (Alrahahleh et al., 2017; Heinonen et al., 2018); therefore, we here also present the impact of the interaction between the CCMS and climate change on ES and biodiversity. We study a significant proportion of the boreal region, specifically Sweden, which produces 10% of the wood traded on the global wood market, including 11% of the sawlogs and 25% of the pulp products for the EU28 (FAO, 2021).

The aim of this study is to investigate whether different CCMS will change the future supply of forest ES, including biodiversity (Figure 1). Specifically, we ask the following questions: (1) Will different CCMS, assuming different wood demand for the 21st century, have different impacts on the overall future forest carbon stock in living biomass and soil (“regulating ES”) and “non‐wood ES” (Table 1), and how will the CCMS change ES multifunctionality? (2) How will climate change affect the future supply of “wood ES” and “non‐wood ES,” and further also ES multifunctionality? (3) How will the combination of different CCMS (assuming different wood demand) and climate change affect future ES levels and multifunctionality? Finally, (4) how will these CCMS and future climates change local synergies and trade‐offs between ES supply, that is, the capacity of the forest to supply multiple ES?

## 2 METHODS

### 2.1 Study area and forest projections

For the Swedish boreal and hemiboreal zone, we projected forest dynamics, management, and ES levels for all the combinations of four scenarios implementing different CCMS (*Current Policy*, *Bioenergy*, *Bioeconomy*, and *Set‐aside* scenario) and three climate scenarios (Constant Climate [CC], Representative Concentration Pathway [RCP]4.5, and RCP8.5). We projected the levels of ES on 29,892 plots of the Swedish national forest inventory (NFI), which represent all productive forest in Sweden (the 23 million ha producing ≥1 m^3^ of wood ha^−1^ year^−1^, corresponding to 1.4% of the global boreal biome), including production and protected land (Fridman et al., 2014). The projections were initialized with observed levels for wood ES and the model‐predicted levels for non‐wood ES based on data from 2008 to 2012 (“2010” henceforth). Projections were made for the period 2010–2100; results were analyzed from 2020, the year of the first GHG mitigation target of the *Current Policy* scenario.

Forest dynamics and management were projected with the Heureka system (http://www.slu.se/en/sha, Wikström et al., 2011). The Heureka core contains a set of empirical growth and yield models that simulate the development of the tree layer in 5‐year time steps, including models for stand establishment, diameter and height growth, ingrowth, and mortality. Climate change modifies tree growth based on the process‐based vegetation model BIOMASS (McMurtrie & Wolf, 1983), indirectly implemented as an approximation model (Eriksson et al., 2015). Decomposition is modeled by the dynamic soil carbon Q‐model, a cohort‐based decomposition model that follows the mass loss of litter over time for different litter compartments. The Heureka application PlanWise allows to determine the optimal combination of management strategies that meet user‐defined objectives and constraints. For each NFI plot and time step, a large number of management activities (such as thinning and clear felling) are simulated, that in sequence constitute different treatment schedules. In a harvest scheduling model (Johnson & Scheurman, 1977), the optimal treatment schedule for each plot is selected based on an objective function and possible constraints using a built‐in optimization tool based on the ZIMPL optimization modeling language (Koch, 2014).

### 2.2 ES including biodiversity

We studied seven ES that can be classified according to the Common International Classification of Ecosystem Services and Nature's Contributions to People (Table 1). The ES were net biomass accumulation (kg m^−2^ year^−1^), defined as change in wood stock between consecutive 5‐year time steps excluding the biomass accumulation of trees that have been felled or died, carbon storage in living trees (kg m^−2^), carbon storage in soil (kg m^−2^), deadwood volume (m^3^ ha^−1^), bilberry plant cover (%), wildfood plant cover (%), and understory plant species richness. Deadwood volume and understory species richness represent biodiversity (see Gamfeldt et al., 2013; Jonsson et al., 2019, 2020 for detailed definitions).

The four first ES are output variables from Heureka. As models predicting the supply of the last three ES as a function of both explanatory forest and climate variables were lacking, we fitted such models. The resulting ES models were then used to project the non‐wood ES on the NFI plots (details in paragraph 2.5 and in Appendix S1).

### 2.3 Scenarios of CCMSs

Projections of demand of wood for material and energy purposes for CCMS scenarios were sourced from the GLOBIOM partial equilibrium model (Havlik et al., 2015; Lauri et al., 2017). GLOBIOM is designed to investigate the impacts of policies concerning the use of biomass, resource efficiency, timber harvesting, the forest‐based industry sector, bioenergy development, and land use development. For this study, we applied the EU version of the GLOBIOM model, where the globe is represented at the level of 58 geographic regions which are connected through bilateral trade flows (28 EU Member States and 30 regions outside the EU). For each of these regions, GLOBIOM simulates the future development of the forestry, agriculture, and bioenergy sectors and provides Heureka with a projection of the future wood demands for Sweden. GLOBIOM has a long history of usage for investigating impacts of EU policies on bioeconomy and climate change mitigation in terms of their direct and indirect impacts on production and consumption of wood materials, international trade and future harvest levels in different countries (Capros et al., 2013; European Commission, 2018; Forsell et al., 2016; Nordström et al., 2016; extended description in paragraph 1.1 in Appendix S2).

We projected three CCMS scenarios using GLOBIOM. These scenarios led to different projected wood demand from Swedish production forest, that is, the 96.4% of the forest area producing >1 m^3^ ha^−1^ year^−1^ of wood (see Section 3 below). The fourth *Set‐aside* scenario presents developments of the ES on the forest area set‐aside in reserves (see below). The scenarios consider energy policy targets, regulation, as well as 2030 and 2050 targets for climate change mitigation of the EU. Further EU‐level developments considered are the bio‐based economy and changes in end‐user consumption patterns affecting wood demand. For all non‐EU countries, the assumptions remain consistent between the scenarios.

#### 2.3.1 Current Policy scenario

A CCMS scenario that implies a EU‐wide 20% reduction in GHGs by 2020, compared to the 1990 emission level, and thereafter no further climate change mitigation actions. This scenario is in line with the official 2013 EU Reference scenario (Capros et al., 2013) and considers a broad range of policy commitments, currently implemented policies, legislations and targets adopted by the individual EU Member States as well as at the EU level. Key policies for the EU accounted for in this scenario include the Renewable Energy Directive (2009/28/EC), the Energy Efficiency Directive (2001/27/EU), and the GHG Effort Sharing decision (No 406/2009/EC). From 2012 onwards, no changes in policies are assumed and no new policies are considered.

#### 2.3.2 Bioenergy scenario

A CCMS scenario where more climate change mitigation action is taken to further reduce GHG emissions within Europe. In this scenario, the wood demand for bioenergy in the EU28 Member States reaches levels that are consistent with an EU‐wide GHG emission reduction of 40% by 2030 and 80% by 2050 compared to 1990 (European Commission, 2012; Frank et al., 2016).

#### 2.3.3 Bioeconomy scenario

A CCMS scenario including the targets of *Current Policy* and *Bioenergy* scenarios, but also with an additional 60% reduction in global GHG emissions by 2050 compared to the 2010 level. This scenario assumes a stronger development of the bioeconomy and resulting demand of bioproducts such as biomaterials, biochemicals, and biofuels. In particular, the impact of an increased demand for material with a high substitution for materials nowadays produced from crude oil and natural gas has been considered. The projection outlines the possible development of several product types, such as polymers, solvents, detergents, and lubricants to not only cover wood‐based material but also the full range of possible bio‐based products.

#### 2.3.4 Set‐aside scenario

In addition to the three CCMS scenarios for the production land (96.4% of the area), we projected a fourth *Set‐aside* scenario representing NCS. This followed the development on the 3.6% of the productive forest land (specifically, NFI plots) that were protected in nature reserves at year 2010 and were subsequently left unmanaged.

In the projection of the three main CCMS scenarios on production land, wood demands projected by GLOBIOM were specified as constraints in the optimization of the future national‐scale forest management on the NFI plots. For each plot, a number of management regimes was projected (paragraph 1.2 in Appendix S2). In the optimization, a combination of these management regimes (i.e., a treatment schedule) was chosen that maximized net present value (NPV) while satisfying the wood demand. NPV is defined as the economic outcome of present and future forestry activities, that is, predicted future income minus cost for future activities such as thinning, clear‐cutting, and appropriate regeneration from period 1 to infinity, discounted back to the present with a 2.5% interest rate (Eggers & Öhman, 2020). Managing the forest to maximize NPV means applying to all NFI plots on production forest, a combination of management regimes that gives more importance to the current economic value of the harvested wood than to its future value.

### 2.4 Climate scenarios

The CCMS scenarios and resulting forest dynamics and management were projected under three climate scenarios. The first one was CC, assuming that averages from the period 1983–1992 remained constant for the whole projection period. This is the period from which the Heureka growth models were calibrated (Fahlvik et al., 2014). The other two scenarios were the two IPCC RCPs (van Vuuren et al., 2011): RCP4.5 and RCP8.5. Climate projections based on RCP4.5 assume a moderate GHG emission reduction consistent with current emission trajectories and policy commitments (2–4.5°C increase by 2100 for Sweden), and RCP8.5 assumes no emission mitigation undertaken (4–7°C increase for Sweden). To predict non‐wood ES, we extracted the climate predictors temperature and precipitation sums for the projection period at the coordinates of NFI plots following the procedure in Mair et al. (2018; paragraph 2 in Appendix S2).

### 2.5 Bayesian GLMs for non‐wood ES predictions

The projections of the three non‐wood ES were predictions from the fitted Bayesian Hierarchical GLMs for each plot in each time step. Projections of the predictors of non‐wood ES came from three sources: the tree age and biomass data were derived from Heureka projection outputs; the climate predictors from the regionally downscaled climate projection data from the Swedish Meteorological and Hydrological Institute also used for model fitting (see Section 2.4); soil moisture from the NFI data (assumed constant over the projection period). The procedures for fitting the Bayesian GLMs, description and selection of predictors, parameter descriptions, prior distributions, and predictive performance are reported in Appendix S1.

### 2.6 Effects of climate and CCMS scenarios on ES and ES multifunctionality

We tested the effect of CCMS and climate scenarios, separately and in combination, on the mean value of single ES among all NFI plots in each time step and on ES multifunctionality. Plot‐level ES multifunctionality, the potential of each NFI plot to supply multiple ES, was calculated by summing the levels of the seven ES. To account for the different measurement scales, each ES was scaled to values between 0 and 1 (*Z*) in relation to the range of values observed (*x*) among all plots and all time steps as,

$$Z=\left(x-\min\left(x\right)\right)/\left(\max\left(x\right)-\min\left(x\right)\right).$$

This ES multifunctionality measure assumes that each ES has equal importance, and that each increase in ES level has the same importance in terms of benefits provided (following Manning et al., 2018). This is a fairly simple multifunctionality measure (i.e., in reality not all services are equally valued by society) that serves well for a quick comparison of overall service provisioning among the tested scenarios. However, energy and products from wood has a strong weight in the scenarios as the management maximizes NPV.

We tested the effect of climate versus CCMS scenarios and their interactions on levels of ES and ES multifunctionality with GLMs (details in Appendix S3). We tested for scenario effects at the beginning (2020), the middle (2060), and the end (2100) of the projection horizon and reported the average values (Tables S3 and S4).

### 2.7 Change in future ES synergies and trade‐offs

We quantified future changes in relationships between pairs of ES across the entire country (i.e., the NFI plots) by calculating the difference in Pearson correlations between the beginning and the end of the projection horizon, 2020 versus 2100. We investigated the effects of climate change and CCMS scenarios separately in production forest and the *Set‐aside* scenario (Figure 4; Table 1). Synergies between pairs of ES were defined as positive correlations (*R* > 0) and trade‐offs as negative correlations (*R* < 0).

The projections of the non‐wood ES into the future, the statistical analyses, and plots were all produced using R version 3.6.2 (R Core Team, 2019). The Bayesian GLMs were fitted using MultiBUGS (Goudie et al., 2020) based on the BUGS program (Gilks et al., 1993).

## 3 RESULTS

### 3.1 Impact of CCMS on future ES levels

**FIGURE 2:**
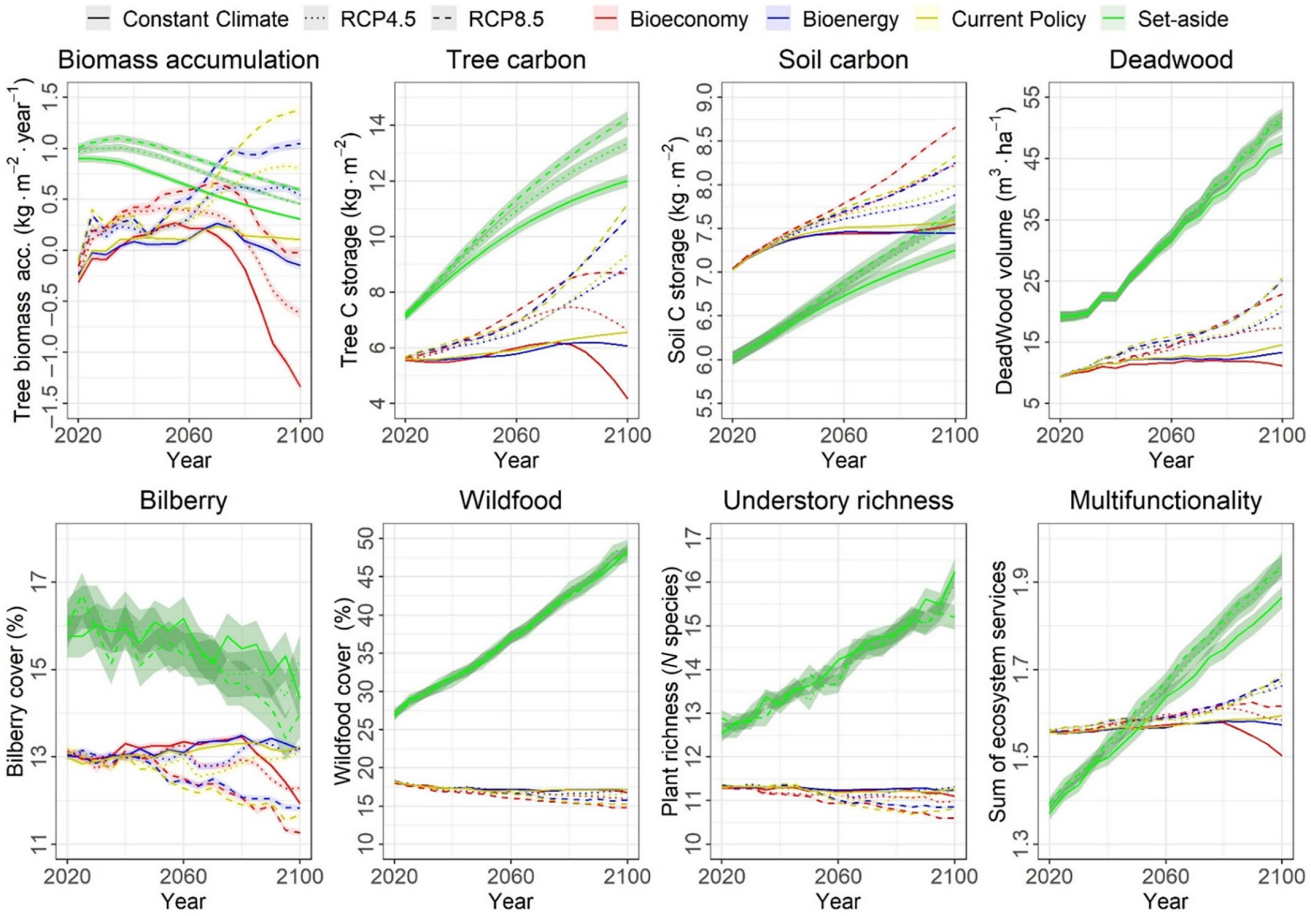
Projected mean levels and standard errors of seven ecosystem services and multifunctionality for the Swedish forests given by four climate change mitigation solutions and three climate scenarios. RCP, Representative Concentration Pathway.

Throughout the projections, the levels of almost all the ES, except for soil carbon, were noticeably lower in production forests than in the *Set‐aside* scenario (Figure 2). In the production forests, the levels of the four wood ES and ES multifunctionality all increased but at different rates in the first 40 years of the projection (2020–2060). In the last 40 years of the century (2060–2100), the levels of wood ES and ES multifunctionality generally continued to increase, but did so the least in the *Bioenergy* and *Bioeconomy* scenarios with increased wood demand. In contrast, the levels of non‐wood ES in production forests decreased at different rates along the 21st century. They decreased less under the *Bioenergy* scenario and more under the *Bioeconomy* scenario compared to the *Current Policy*.

In the *Set‐aside* scenario (Figure 2), the level of ES multifunctionality and almost all ES increased along the 21st century, with the exception of net biomass accumulation and bilberry cover, which slightly decreased compared to their initial levels. In *Set‐aside*, the trends of ES levels were unaffected by CCMS scenarios, as these forests were not harvested.

**FIGURE 3:**
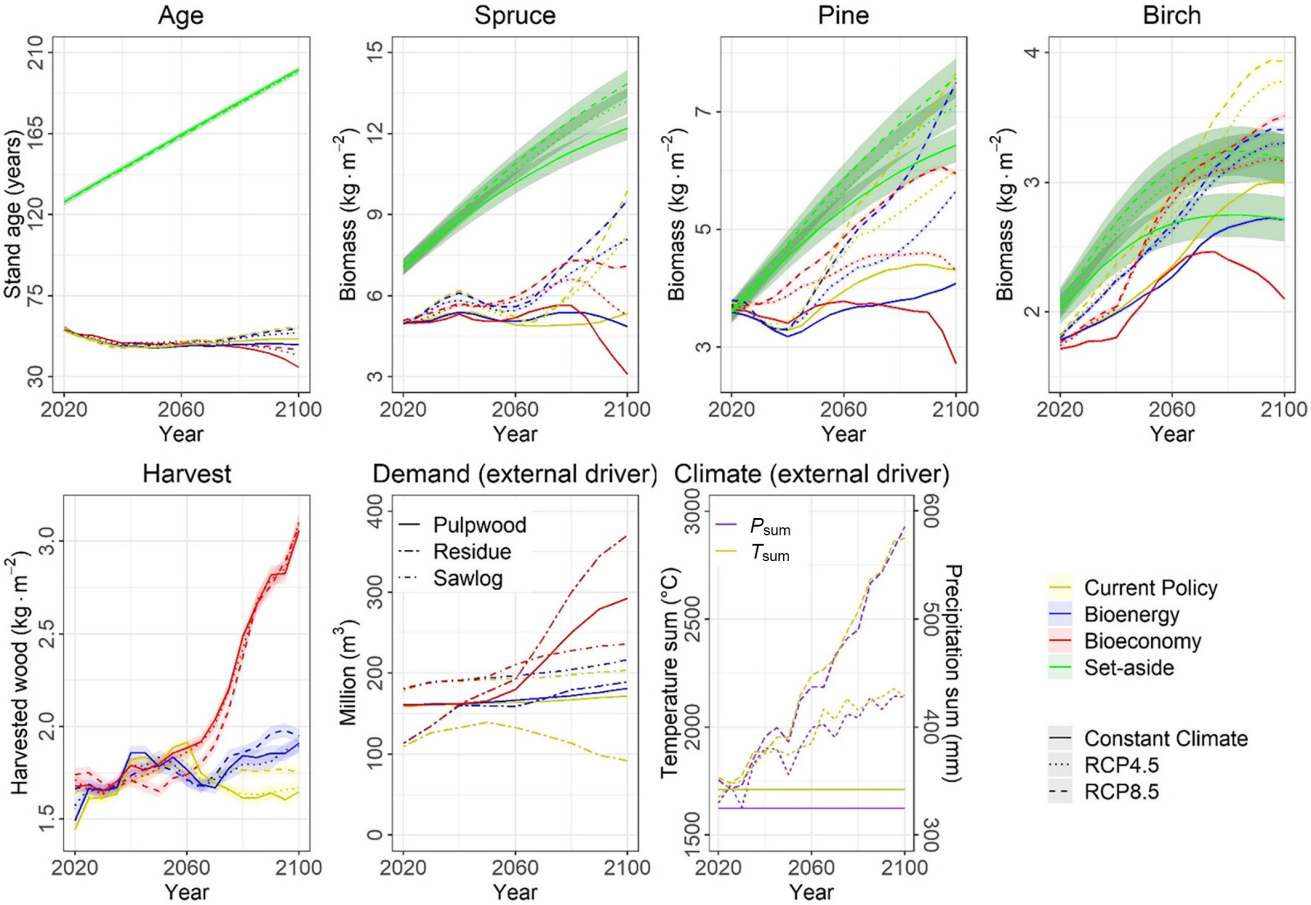
Projected mean levels and standard errors of predictors and drivers of ecosystem services for the Swedish forests given by four climate change mitigation solutions and three climate scenarios. Temperature (*T*_sum_) and precipitation (*P*_sum_) sums are calculated over the growing season. RCP, Representative Concentration Pathway.

Net biomass accumulation in production forests increased in the first 40 years (2020–2060) in all CCMS scenarios (Figure 2; Table S3). This general increase occurred in parallel with a moderate increase in timber harvesting (5%–33% under the *Current Policy* scenario, 3%–8% under the *Bioenergy* scenario and 0%–12% under the *Bioeconomy* scenarios), which caused a moderate decline in stand age (Figure 3). In the last 40 years (2060–2100), net biomass accumulation remained stable or even increased under the *Current Policy* and *Bioenergy* scenarios, with a smaller increase in the latter than the former, but declined under the *Bioeconomy* scenario (Figure 2; Table S3). These differences between scenarios in 2060–2100 occurred in parallel with increasing timber harvesting after 2060 to meet the increasing demand of wood for bioproducts and bioenergy entailed by the different CCMS scenarios (Figure 3). Consequently, while in *Current Policy* and in *Bioenergy* stand age remained stable or increased, it dramatically decreased under the *Bioeconomy* scenario resulting in low further net biomass accumulation (Figure 3).

Carbon storage in trees and soil increased in production forests irrespective of the CCMS scenario in years 2020–2060 (Figure 2). In 2060–2100, the C storage further increased under *Current Policy*, but less so under *Bioenergy*. In *Bioeconomy*, C storage decreased in parallel to net biomass accumulation. This lower accumulation of C in trees and soil is caused by the harvest increase under the *Bioenergy* (10%–14%) and *Bioeconomy* scenarios (70%–77%) reducing tree stocks and soil litter accumulation (Figures 2 and 3; Table S2).

Deadwood and non‐wood ES in production forests were not significantly different among CCMS scenarios in years 2020–2060 (Figure 2; Table S3). However, during 2060–2100 when comparing *Bioeconomy* to *Current Policy*, deadwood increased somewhat less and non‐wood ES decreased instead of remaining stable (Figure 2; Table S3). These lower future levels under *Bioeconomy* were due to the exponential increase in timber harvesting decreasing forest ages and tree stocks (Figure 3) which produce little deadwood and predict low non‐wood ES (Table S1). However, the *Bioenergy* scenario did not markedly reduce the stand age or tree stocks of production forests (Figure 3), hence keeping non‐wood ES at levels comparable to or higher than under *Current Policy*.

Finally, ES multifunctionality increased less under *Bioenergy* and even decreased under *Bioeconomy* compared to *Current Policy* (Figure 2).

### 3.2 Impact of climate change on future ES levels

On the one hand, net biomass accumulation, C storage in trees and soil, deadwood and ES multifunctionality increased with increasing GHG concentrations. Specifically, RCP4.5 and RCP8.5 scenarios projected higher supply of these ES compared to CC (Figure 2; Table S3). On the other hand, increasing GHG concentrations slightly decreased non‐wood ES (Figure 2; Tables S3 and S4). The increases in wood ES and ES multifunctionality were both caused by the increase in stand age and tree stocks under RCP4.5 and RCP8.5 (Figure 3). The same environmental changes instead decreased non‐wood ES.

### 3.3 Joint impact of CCMS and climate change on future ES levels

Wood ES and ES multifunctionality increased with increasing GHG concentrations more in production forest than in the *Set‐aside* scenario (Figure 2; Tables S3 and S4). The positive effect of increasing GHG concentrations from CC to RCP4.5 or RCP8.5 on wood ES and ES multifunctionality further reduced their decreases under the *Bioenergy* and *Bioeconomy* scenarios (Figure 2; interaction terms in Table S3). On the other hand, non‐wood ES significantly decreased with increasing GHG concentrations only in production forest but not in the *Set‐aside* scenario (Figure 2; Tables S3 and S4).

### 3.4 Impacts of CCMS and climate on future ES synergies and trade‐offs

**FIGURE 4:**
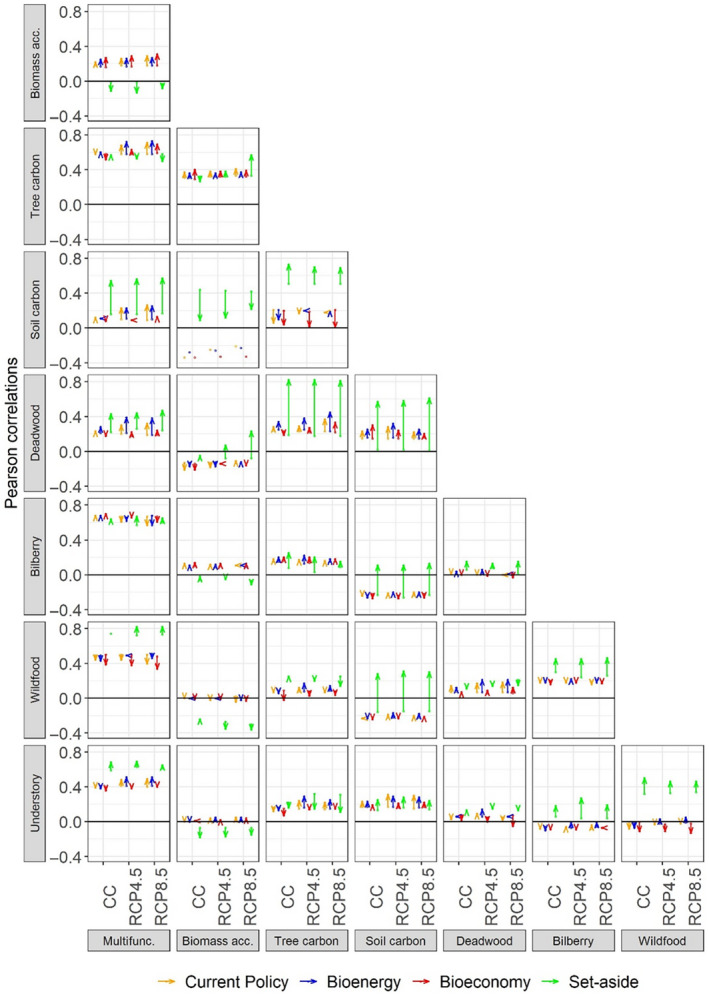
Changes in pairwise correlations between ecosystem services from 2020 to 2100 for Swedish forests given four climate change mitigation solutions and three climate scenarios. Positive and negative correlations reflect synergies and trade‐offs, respectively. Arrows represent start (2020) and end (2100) correlations, so downward arrows represent decreases across time, upward represent increases, and horizontal ones represent stable correlations. CC, Constant Climate; RCP, Representative Concentration Pathway.

Local plot‐level trade‐offs between pairs of ES increased over time with a similar frequency (11%) in the *Set‐aside* scenario and in production forests from 2020 to 2100, but they decreased more frequently in production forests (7%) than in *Set‐aside* (4%, Figure 4). This was independently of which CCMS scenario simulated. Moreover, local synergies between pairs of ES increased more frequently (57%) and decreased less frequently (18%) in *Set‐aside* than in production forests (synergy increase: 44%, synergy decrease: 28%). Finally, a considerable proportion of local trade‐offs became synergies only in *Set‐aside* (10%). For example, the CCMS in production forests maintained stable trade‐offs between regulating (soil C storage) and non‐wood ES (bilberry and wildfood plants) and among non‐wood ES (bilberry vs. understory richness). However, NCS in *Set‐aside* turned trade‐offs between regulating and non‐wood ES into synergies and increased synergies among non‐wood ES (Figure 4).

There were negligible differences between the proportion of local trade‐offs becoming synergies under the climate change scenarios (for RCP4.5 and RCP8.5: 3%) compared to under CC (2%, Figure 4). However, local synergies increased less (RCP8.5: 41%) but decreased more frequently (RCP8.5: 34%) under the highest GHG concentration, compared to climate scenarios with lower GHG concentration (increase CC: 49%, increase RCP4.5: 51%, decrease CC: 25%, decrease RCP4.5: 18%). Consequently, local trade‐offs between pairs of ES decreased less (RCP8.5: 1%) and increased more frequently (RCP8.5: 15%) under high GHG concentration compared to climate scenarios with lower GHG concentration (decrease CC: 9%, decrease RCP4.5: 8%, increase CC: 9%, increase RCP4.5: 10%).

## 4 DISCUSSION

For the first time to our knowledge, we provide an integrated analysis of the joint impact of CCMS and increasing GHG concentrations on future wood and non‐wood ES for a major wood producer for the international market. We show that (1) with bioenergy and bioeconomy solutions the associated increasing demand for wood will decrease ES multifunctionality, but these negative effects will be partially offset by the increasing stand age and tree stocks induced by increasing GHG concentrations. (2) Climate change will have a larger negative impact on the long‐term supply of ES in production forests, where bioenergy and bioeconomy solutions are applied, than in forests protected in set‐asides as part of NCS. (3) Bioenergy and bioeconomy solutions and increasing GHG emissions will cause net decreases in ES synergies and net increases in ES trade‐offs, while NCS will increase the supply of multiple ES in synergy, even turning ES trade‐offs into synergies. (4) Finally, protecting forests as NCS can be considered an adaptation measure to offset the landscape level negative effects of increasing GHG concentrations on the future supply of non‐wood ES.

This means that synergistic and increasing supply of harvested wood, climate regulation, deadwood and non‐wood ES will depend on forest strategies integrating more NCS (protected forest) along with bioenergy and bioeconomy solutions. This integration follows de‐growth scenarios to reach climate targets, instead of technological, negative emission solutions (Keyßer & Lenzen, 2021).

### 4.1 Impact of CCMS on future ES levels

The moderate increase in wood demand given by the *Bioenergy* scenario could be met by an increasing net biomass accumulation derived by a moderate increase in forestry intensity. However, when meeting the increasing demand in the *Bioeconomy* scenario, the long‐term net biomass accumulation dropped because of an excessive wood harvesting. Our findings thus contrast with Nordström et al. (2016), who concluded that we could meet the high demand resulting from ambitious CCMS with current forest management practices. Instead, our finding of a long‐term production drop agrees with conclusions from simulating high intensity harvest across Finland by Heinonen et al. (2017).

The *Bioenergy* and *Bioeconomy* scenarios mean increasing wood demand decrease the supply of regulating ES. This contrasts Lundmark et al. (2014) arguing instead that more intensive silvicultural methods can increase biomass production in Sweden and therefore be an effective way to reduce carbon emissions via increased biomass production, which leads to increased carbon sequestration and the carbon finally stored in wood products. On the other hand, several studies have instead concluded that intensifying biomass removal from forests induces a reduction of carbon accumulated in trees and soil (Achat et al., 2015; Repo et al., 2015; Seppälä et al., 2019; Soimakallio et al., 2021). Bioenergy or bioeconomy solutions, as advocated by Lundmark et al. (2014), have indeed been criticized for, at least initially, exacerbating rather than mitigating climate change (Norton et al., 2019). We confirm that the intensive harvesting under a *Bioeconomy* scenario, including a large proportion of even‐aged management with slash and stump removal, is in conflict with carbon storage, as was also concluded in a recent EU report on the use of biomass for energy production (Camia et al., 2021). Overall, an increased use of biomaterials may create a forest carbon debt that is not compensated for within a decadal time horizon, if the biomaterial lifetime is short in relation to the rate of forest carbon regeneration (Seppälä et al., 2019) and depending on the fossil energy/material substituted and the emissions during harvesting (Soimakallio et al., 2021). Generally, long‐lived products have the potential to contribute to climate change mitigation by increasing the carbon storage in harvested products, but that takes time, thus they may not do so in the short term (Taeroe et al., 2017). Finally, the conclusion relies on high substitution factors, which is not applicable if consistent climate mitigation policies are to be implemented (Skytt et al., 2021).

The reason why CCMS based on increasing wood demand caused a decrease in deadwood and non‐wood ES is the resulting parallel decline in mean stand age and biomass stocks of the main tree species. These are key variables determining the levels of these ES, and with high demands for wood the necessary old, mixed, high‐volume forests decline (Gamfeldt et al., 2013; Jonsson et al., 2019; Table S1).

The explanations for the continuous future increase in almost all ES within protected forests (NCS) are all clear. Our results demonstrate that, although protected forests (NCS) have slower biomass growth than production forests managed for bioenergy and bioeconomy solutions, their mature trees still store more carbon contributing to regulating ES, confirming projections from process‐based models (Augustynczik & Yousefpour, 2021). Thus, stopping harvesting in the set‐asides to allow forests to mature and carbon stocks to increase can be an efficient CCMS. Large‐scale natural disturbances, like the large storm that occurred in Sweden in 2005, can indeed cause a sharp decrease in the carbon stock of the forests earlier sequestering carbon (Lundmark et al., 2014). However, there is potential to increase the resilience of the forest to future storms by adopting management regimes that mean approaching the structure of protected forests, like mixed species or continuous cover forestry (Hahn et al., 2021). Moreover, these regimes could also increase the supply of deadwood and non‐wood ES and are advocated in both the EU Biodiversity strategy for 2030 (European Commission, 2021b) and the New EU Forest Strategy for 2030 (European Commission, 2021c). Also, the future ES multifunctionality increased more in forests protected for NCS than in forests managed for bioenergy and bioeconomy solutions, in agreement with recent projections by Pukkala (2022) and empirical data on relationships between forest age and ES levels (Table S1; Jonsson et al., 2020). Pohjanmies et al. (2021) also found that protection should be the dominant NCS to optimize ES multifunctionality. Thus, to increase the area of multifunctional forest, the proportion of the protected landscape must increase, for example, reaching the targets of the Biodiversity Strategy (European Commission, 2021b) or the Aichi target 11 of the Convention on Biological Diversity.

### 4.2 Impact of climate change on future ES levels

We show that increasing GHG concentrations will enhance tree stocks and carbon storage, resulting from increased wood increment projected under RCP4.5 and RCP8.5 (Poudel et al., 2011; Subramanian et al., 2019). This leads to larger deadwood amounts, in agreement with local projections for southern and northern Finland under IPCC SRES A2 (Mazziotta et al., 2014). We further found that this resulting increasing tree stocks will reduce the supply of the non‐wood ES associated with ground floor vegetation, in agreement with long‐term monitoring and experimental data for Sweden and explained by the increased shade of dense canopies (Hedwall et al., 2019; Strengbom et al., 2018).

We provide support for increasing ES multifunctionality with increasing GHG concentrations. Indeed, climate change will decrease non‐wood ES, but it is not the sole factor driving ES multifunctionality. The mechanism behind our findings is that RCP4.5 and RCP8.5 means longer growing seasons (determined by increasing CO_2_ and temperatures over longer periods), accelerating the rates of all forest processes (tree growth and litter decomposition) providing ES (Eriksson et al., 2015; Mazziotta et al., 2014).

Projections of forest developments into the future have different uncertainties affecting projected ES levels (Thom & Seidl, 2016). For example, an increase in storm intensity may reduce the increase in tree stocks by the end of the 21st century (Subramanian et al., 2019). Increasing wind intensities may further increase the profitability of non‐typical management regimes (Hahn et al., 2021). The warming should increase in the intensity and frequency of wildfire that will impact the future supply of regulating ES, and may even switch the role of forests from carbon sink to carbon source (Lidskog & Sjödin, 2016). Potentially increasing frequency of outbreaks of insects (Hof & Svahlin, 2016) and pathogens (Sipari et al., 2022) should increase tree mortality and hence deadwood dynamics and quantity (Mazziotta et al., 2014). Finally, the current version of Heureka most likely underestimates the effect of future extreme events, such as droughts. The warming‐induced increases in evapotranspiration can actually decrease growth in significant parts of the country (south and east, Belyazid & Zanchi, 2019). There is therefore a risk that the increase in tree stocks is overestimated. However, the relative contribution in how these natural and technical uncertainties plays out in the future, individually, in combination, on small or large scales is highly uncertain.

### 4.3 Joint impacts of CCMS and climate change on future ES levels

We show that climate change is likely to alter the long‐term supply of ES more strongly in production forests than in protected ones. Specifically, increasing GHG concentrations will have negative impacts on non‐wood ES in production forests, but not in protected ones because of the dampening, contrasting effect of increasing tree age. Furthermore, increasing GHG concentrations can partly offset the negative effects of bioenergy and bioeconomy solutions on wood ES and ES multifunctionality. Thus, protection via NCS is a buffer option decreasing the negative impact of increasing bioenergy and bioeconomy solutions and GHG concentrations on non‐wood ES, ultimately increasing forest resilience to climatic change (Chapin et al., 2007).

### 4.4 Impacts of CCMS and climate on future ES synergies and trade‐offs

Bioenergy and bioeconomy solutions relying on increasing wood harvest and future climate change will modify the capacity of forests to simultaneously supply multiple ES, here shown as changing future synergies and trade‐offs. Increasing tree stocks with increasing GHG concentrations will supply more wood (Baul et al., 2017; Gustavsson et al., 2017) and if increasingly harvested it will lead to net decreases in ES synergies (less frequent increases and more decreases), and net increases in ES trade‐offs (more frequent increases and fewer decreases). This points to decreasing uses of individual forest stands in the future, potentially increasing the conflicts in the use of the forest also for climate change mitigation and biodiversity. There will thus be a continued conflict resulting from policy focusing on wood ES versus on regulating ES, deadwood and non‐wood ES, the latter also reflecting biodiversity. Specifically, there will be decreasing synergy between tree and soil carbon, and further persistent trade‐offs between net biomass accumulation and soil carbon, deadwood, wildfood plant cover, and understory plant species richness. It has previously been found that increased extraction of provisioning ES (e.g., timber) creates a trade‐off with future supply of non‐provisioning services in boreal Canada (Erdozain et al., 2019) and that targeting high timber revenues or bioenergy with increasing timber harvesting poses trade‐offs with deadwood supply and carbon storage in boreal Finland (Díaz‐Yáñez et al., 2021; Triviño et al., 2017) and in Mediterranean forests (Morán‐Ordóñez et al., 2020).

Among the CCMS, only NCS (the *Set‐aside* scenario) will strongly increase the supply of multiple ES in synergy, even turning current ES trade‐offs into future synergies. More specifically, the frequency of synergies will increase between tree carbon and soil carbon, deadwood and bilberry cover, between soil carbon and deadwood, and among all non‐wood ES. There will also be a switch from trade‐off to synergy between net biomass accumulation and deadwood, and between soil carbon and bilberry/wildfood plant cover as forests age undisturbed. This suggests that CCMS based on low impact forestry may stimulate current synergies between multiple ES and further create new environmental conditions with more synergies. NCS may, for example, increase the local alignment between carbon storage (Nordström et al., 2016), accumulation of deadwood for saproxylic species (Bouget et al., 2012) and development of habitat to support high bilberry cover, herbivore forage plants, and high understory plant diversity (Anderson et al., 1969; Eckerter et al., 2019). This increase in synergies between non‐wood ES is likely explained by the complementary micro‐environmental conditions developing in old‐growth, mixed forests allowing for niche differentiation in resource exploitation among high diversity of species providing ES (Williams et al., 2017).

In the Finnish Nordic forests, the forest soil expectation value doubles when berry production is taken into account (Miina et al., 2016). In Europe, the total value of non‐wood forest ES collected each year amounts to 71% of the roundwood production value, but in Sweden their value is marginal (6%–12% of the roundwood production value depending on location and site; Lovrić et al., 2020). Given this economic incentive, forest strategies including CCMS that consider co‐production of wood and non‐wood ES have the potential to achieve joint climate and biodiversity benefits with limited economic losses (Kurttila et al., 2018). Although a review has shown that forestry targeting high levels of ES multifunctionality provides less timber (Sing et al., 2018), recent work has demonstrated that it is possible to reconcile the biomass harvesting targets of a bioeconomy with also a supply of regulating and cultural ES through the principles of Climate‐Smart Forestry (Verkerk et al., 2020). This can be operationalized through a land sparing management, spatially differentiating between areas supplying timber at high rates, and areas devoted to climate change mitigation, non‐wood ES provisioning and biodiversity conservation (in Finland: Eyvindson et al., 2018; Kärkkäinen et al., 2020; Mönkkönen et al., 2014; Triviño et al., 2017; in North America: Côté et al., 2010; Tittler et al., 2012).

### 4.5 Concluding remarks

Even if CCMS based on increasing wood demand are expected to drastically reduce forest multifunctionality, protection via NCS has the potential to buffer against this negative impact, constituting a stronghold to increase the capacity of the forest to supply multiple ES in synergy. Our study has demonstrated that NCS can be included within CCMS to assure that the timber harvesting rate needed to achieve climate change mitigation targets will be sustainable also from the perspective of regulating ES and the maintenance of deadwood and non‐wood ES (Eyvindson et al., 2021; Peura et al., 2018), as also envisioned by the EU Bioeconomy strategy (European Commission, 2018) and the EU Biodiversity and New forest strategies for 2030 (European Commission, 2021b, 2021c). Recently, the “Fit for 55” climate package under the European Green Deal was presented, aiming to reduce GHG emissions by 55% by 2030 compared to 1990 (European Commission, 2021d). To meet these targets, EU member states are developing strategies of Climate‐Smart Forestry (Nabuurs et al., 2007; Yousefpour et al., 2018) to increase biomass production to fulfil higher future demand for bioenergy and bioeconomy (Bioeconomy Strategy, 2018; EASAC, 2017). We show that greater integration of NCS into these strategies will be crucial to increase the synergistic supply of ES under climate change in boreal countries worldwide (Díaz‐Yáñez et al., 2020; Lagergren & Jönsson, 2017).

## CONFLICT OF INTEREST

There are no potential conflicts of interest.

## AUTHOR CONTRIBUTIONS

Adriano Mazziotta and Tord Snäll conceived the study. Adriano Mazziotta and Tord Snäll fitted the models for non‐wood ES and undertook the rest of the analyses. Johanna Lundström and Jeannette Eggers prepared the CCMS scenarios. Adriano Mazziotta and Tord Snäll led the writing of the manuscript. Adriano Mazziotta, Johanna Lundström, Nicklas Forsell, Helen Moor, Jeannette Eggers, Narayanan Subramanian, Núria Aquilué, Alejandra Morán‐Ordóñez, Lluís Brotons, and Tord Snäll interpreted the results and participated in writing the paper.

## Supporting information


Appendix S1

## Data Availability

The data that support the findings of this study are available from the Dryad Digital Repository: https://doi.org/10.5061/dryad.p8cz8w9t5 (Mazziotta et al., 2022).
