# Supplementary material for: More future synergies and less trade‐offs between forest ecosystem services with natural climate solutions instead of bioeconomy solutions
Source: Glob Chang Biol. 2022 Aug 10;28(21):6333–48. doi: 10.1111/gcb.16364 (PMC9805065; doi:10.1111/gcb.16364)
Supplement: Supplementary file 1 — Appendix S1 [file GCB-28-6333-s001.docx]

**SUPPORTING INFORMATION**

**Appendix 1. Bayesian GLMs for non-wood ecosystem services (ES)**

1. **Description of models for three non-wood ecosystem services**

The projections of the modelled non-wood ES levels were obtained as follows: *first*, the relationships between ES and forest/climate covariates were estimated by fitting three separate Bayesian hierarchical Generalized Linear Models (GLMs; Gelman et al., 2004). *Second*, these relationships were used to project ES levels under Climate Change Mitigation Solutions (CCMS) and climate scenarios for all National Forest Inventory (NFI) plots (representing all productive forest) over the 21^st^ century. We fitted hierarchical GLMs to acknowledge the hierarchical higher tract level variability of the climate variables and the lower plot-level variability of the forest variables in the NFI data. The NFI uses a randomly planned regular sampling grid and includes 29,892 permanent and temporary tracts. Each tract has been surveyed once every 5 years. The tracts, rectangular in shape and of different dimensions in different parts of the country, contain from 8 (in the north) to 4 (in the south) circular sample plots (each plot 314 m^2^, Fältinstruktion, 2021).

In the building of the non-wood ES models, we investigated including the following forest related predictors (Table S1): stand age, soil moisture, presence of peat in the parental soil, biomass of the main tree species (spruce, pine, and birch), their squared terms for modelling possible non-linear relationships, and interactions between terms. All the ES and forest predictors were measured at the NFI plots for the years 1999-2002, hereafter referred to year 2000. We further investigated including temperature and precipitation sums affecting the dynamics of the plants forming these non-wood ES. They were defined as the yearly sums of mean daily temperatures and precipitations for the growing season, i.e., for all the days in the year when mean daily temperature was higher than 5 °C for at least four consecutive days, and obtained as grids with a spatial resolution of 5 x 5 km^2^ from the Swedish Meteorological and Hydrological Institute. Starting temperature and precipitation sums for the Constant Climate scenario were calculated as means for the period 1989-2010 based on the RCP4.5 scenario (see paragraph 2.4 for details on the climate scenarios).

The models fitted for the three non-wood ES were different to the models fitted for Gamfeldt et al (2013) in that they only included forest predictors whose dynamics can be projected with the Heureka forest simulator. For example, we excluded pH whose dynamics cannot be projected by Heureka. However, the effect of pH changes through the forest succession was captured indirectly by stand age. Other important predictors that could not be projected but could be assumed constant through the projection period, such as soil moisture, were retained and part of the model selection. We also excluded nitrogen deposition because we lack long-term projections for this variable.

We modelled each non-wood ES as a function of the forest and climate predictors . For each ES, we modelled the mean (*μ*), transformed by the link function *g (),* on plot *p* = 1, …, 8, on tract *t* = 1, …, 1,394 as

(eq. 1) $g\left( \mu_{p,t} \right)=\alpha_{t}+\sum_{n=1}^{N} \beta_{n}X_{p,t}+\epsilon_{p,t}$,

where $X_{p,t}$ is a design matrix of *n* plot-level explanatory variables, and $\beta_{n}$ is a vector of associated effect-size parameters. Predictor variables were centered and standardized for each of the five Swedish NFI regions (Fältinstruktion, 2021). The tract-level intercept parameters $\alpha_{t}$ were modelled as $\alpha_{t}\sim\mu_{\alpha_{t}}$, where $\mu_{\alpha_{t}}=\gamma+\sum_{m=1}^{M} \rho_{m}Z_{t}$. $Z_{t}$ is a design matrix of *m* = 1, …, M ≤ 2 tract-level, centered and standardized explanatory predictors (Tsum and Psum), $\rho_{m}$is a vector of associated effect-size parameters, and $\gamma$ is an intercept parameter.

The presence-absence of bilberry was modelled with a Bernoulli distribution, with a mean (*μ*) and a logit link function. The values of bilberry cover and wildfood plant cover larger than 0 were assumed to follow beta distributions (logit link function), with two shape parameters phi (*φ*) and mean (*μ*), and residual contributions $\epsilon_{p,t}$, where $\epsilon_{p,t}$ ~ N(0, *σ*), which means normally distributed with mean = 0 and s.d. = σ.

The values of understory plant richness were assumed to follow a Poisson distribution (log link function) with overdispersion contributions $\epsilon_{p,t}$ ~ N(0, *σ*).

The final model for each non-wood ES was selected based on the Deviance Information Criterion (DIC, Spiegelhalter et al., 2002), on the posterior distribution of the effect size parameters ($\beta_{n}$ and$\rho_{m}$), and on knowledge of the biological system studied. We first assessed the predictive power of each predictor variable separately based on the DIC and on the posterior distributions of $\beta_{n}$ and$\rho_{m}$. Next, we fitted a full model containing the retained predictor variables. Finally, we simplified this full model with a backward stepwise procedure. Parameter estimates (means and ranges of the posterior distributions) for the final model for each ES are presented in Table S1.

We assumed uninformative prior distributions for all parameters (Table S2). The models were fitted using the software MultiBUGS (Goudie et al., 2020) based on the BUGS program (Gilks et al., 1993).

**Table S1.** Estimates of parameters associated with predictors in the final model for each non-wood ES. The means and the outer limits of 95% Bayesian confidence intervals (in parentheses) of the posterior distributions of the parameters ($\beta_{n}$, $\rho_{m}$ $\gamma$ and φ) are shown for each ES (bilberry presence/absence, bilberry cover, wildfood plant cover, understory plant species richness). Terms not included in the ﬁnal main model are labelled as NS (Not Significant).

**Table S2**. Prior distributions for the parameters of the ES models (eq. 1 and following description).

^a^ For understory plant species richness, σ denotes the standard deviation of the plot-level overdispersion contributions.

^b^ For explanatory predictors, see Supplementary Table S1.

^c^ Uniform distribution with minimum 0 and maximum 25.

^d^ Normal distribution with mean 0 and variance 0.001.

^e^ The precision (= σ^-2^) followed a Gamma distribution with shape and scale equal to 0.00001.

We validated the models for non-wood ES by comparing our predictions with the values observed and used for model fitting, specifically NFI data from 2000 as for Gamfeldt et al. (2013). All the three models showed a good level of predictive performance, in terms of variance explained by the model, with a classical R^2^ *sensu* Gelman et al. (2019) equal to 0.82 for bilberry presence/absence, 0.66 for bilberry cover, 0.76 for wildfood plant cover, and 0.54 for understory plant richness. These estimators were confirmed by plots of observed and fitted ES fitted values (Figure S1). However, all the three models showed a limited performance in predicting high and low ES values (Figure S2).

**Figure S1.** Histograms of actually observed and fitted values of non-wood ES.


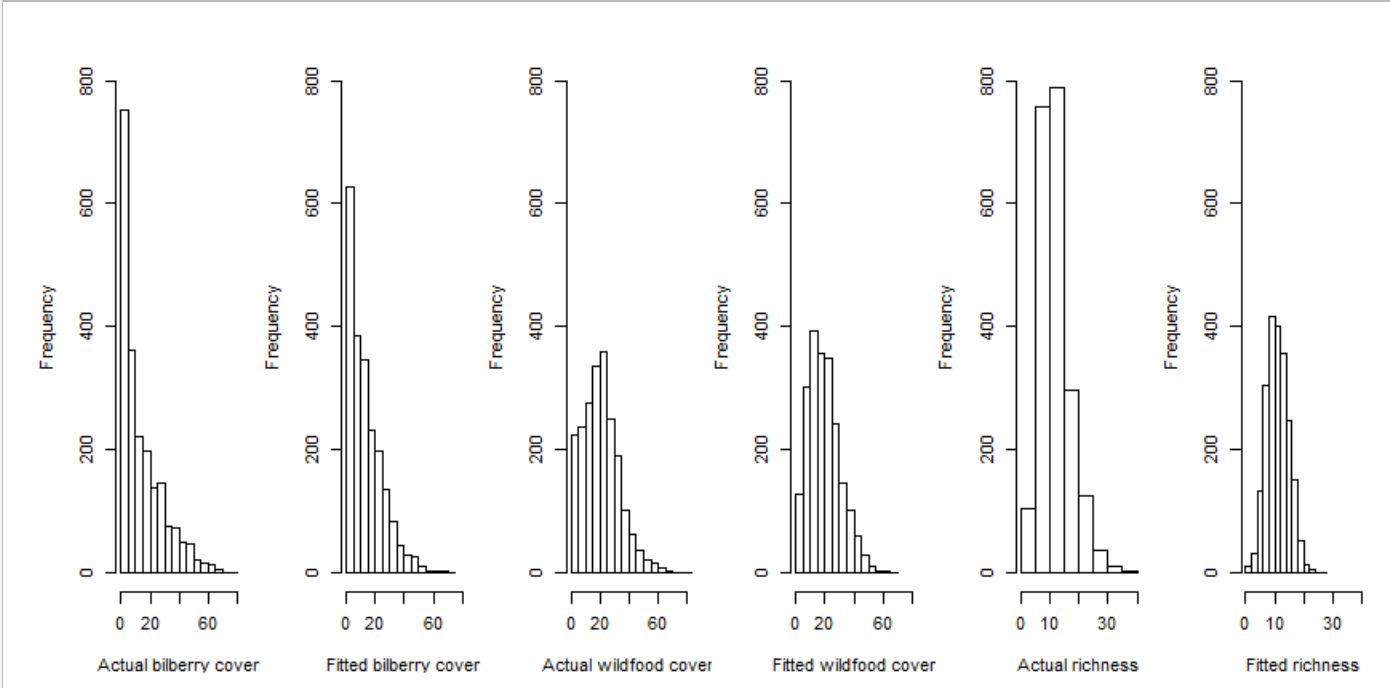


**Figure S2.** Plots of actually observed vs. fitted values (top row) and residuals vs. fitted values (bottom row) using models for non-wood ES models. Red straight lines with equation y ~ x are provided as reference.


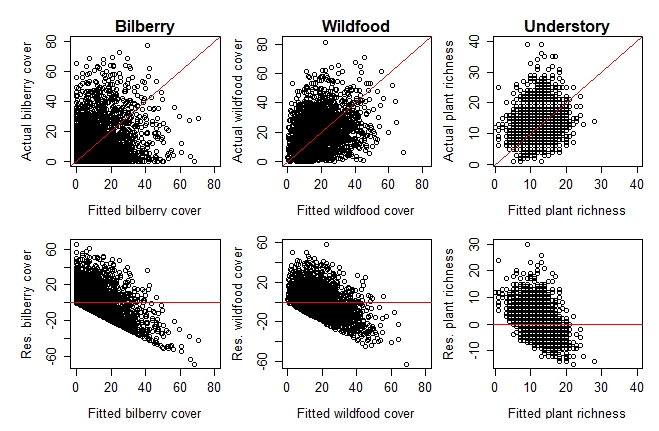


**Appendix 2. Formulation and projection of CCMS and climate scenarios at EU and national scales**

1. **Building the CCMS scenarios**

1.1 The GLOBIOM modelling framework

The quantitative projections of wood and energy use for the European Union countries was conducted using the GLOBIOM integrated modelling framework (Havlik et al., 2015). GLOBIOM is a partial equilibrium model designed to investigate the impacts of policies concerning use of biomass, resource efficiency, timber production, the forest-based industry sector, bioenergy development, and land use development. In essence, it is an economic model that jointly covers the forest, forest industries, bioenergy, agriculture, and livestock sectors, and this allows considering a full range of direct and indirect effects of sustainability policies related to the use of wood and consumption of wood product. GLOBIOM has been used to investigate the impact of EU policies to enhance the bioeconomy and mitigate climate change in terms of their direct and indirect impacts on production and consumption of wood materials, international trade and future harvest levels in different countries.

GLOBIOM has a detailed biophysical basis (G4M and EPIC) which ensures that the processes of biomass production, and environmental and sustainability constraints are accounted for in terms of their effects on forests. GLOBIOM further has a state-of-the-art representation of the forest industries, demand representation of wood categories, and the bioenergy sector allowing for detailed representation of the flow of wood from harvest to consumption and allowing for detailed representation of the development of new products. GLOBIOM has previously e.g., been used to assess the impacts on resource efficiency of future EU demand for bioenergy (Forsell et al., 2016), and the development of the EU level Reference Scenario for the forest and land use sectors (Capros et al., 2013).

1.2 Climate Change Mitigation Solutions scenarios for Sweden

To provide an integrated assessment of the ES provision within Sweden, we couple the GLOBIOM model with that of the Heureka model via demand of wood. We first use GLOBIOM to project the national forest wood demand level for Sweden for each CCMS scenario. The national demand of wood is then used as an input to the Heureka model to project the detailed development of the forests within Sweden. Within Heureka, the three EU-level *Climate Change Mitigation Solutions* (CCMS) scenarios described in paragraph 2.3 of the main text were projected for the Swedish forests through a combination of management regimes in variable proportion that aim to maximize Net Present Value (NPV) from timber extraction. At national level, forests are managed for maximizing Net Present Value (NPV) from timber extraction and with the constraint to deliver the wood demanded given by the three CCMS scenarios according to GLOBIOM.

Using the Heureka system, forest dynamics and management were projected years 2010 to 2100 in five-year time steps for the productive forest for all of Sweden, represented by the NFI plots. The projections were started with the forest conditions observed year 2010 according to the NFI. Alternative development of the forest was projected in two steps. First, several alternative management regimes were generated for each spatial unit (in our case, NFI plots). Second, the most appropriate management regime for each spatial unit, based on the overall objective function and specified constraints was selected using the system’s built-in optimization tool. We specifically maximized NPV, constrained by wood demand given by GLOBIOM.

The ES net biomass accumulation, carbon storage in trees and soil, and deadwood volume are output variables from Heureka. For calculating the ES bilberry cover, wildfood plants for game and plant species richness we instead applied the ES models fitted according to section Appendix 1.1 above and with the predictors stand age, soil moisture, presence of peat in the parental soil, biomass of the main tree species (spruce, pine, and birch) output by Heureka.

Eight management regimes were projected: Business As Usual (BAU: even-aged management, 10 retained trees/ha, 3 high stumps/ha, biofuel extraction at final felling, breeding is used); BAU Focus Bioenergy (like BAU, but biofuel thinning is used); Continuous Cover Forestry (CCF: uneven-aged management, only projected in spruce-dominated forests); BAU Focus Bioenergy Stump Harvest (like BAU, but with extraction of spruce and pine stumps); Promoting Broadleaves (like BAU, but allowing a delay of ten five-year periods in final felling, retaining birch and use them as seed trees, removing conifers at thinning and cleaning); BAU Prolonged Rotation (like BAU, but final felling is allowed 6-10 periods after the lowest allowed final felling age), BAU No Thinning (like BAU, but no thinning treatment) and Set-Aside (No Management).

The BAU regime was most frequently applied under the Constant Climate and under the *Bioeconomy* scenario (Figure S3). BAU Focus Bioenergy was frequently applied under RCP8.5 climate while BAU Focus Bioenergy Stump Harvest was instead more rarely applied in this climate. The application of BAU No Thinning and BAU Promoting Broadleaves was more frequent in RCP8.5, while BAU Prolonged Rotation and CCF were instead applied less frequently in this climate respect to climate scenarios of lower GHG concentrations. The regimes BAU Prolonged Rotation, CCF, and Promoting Broadleaves were frequently applied only under Current Policy.

**Figure S3.** Management regimes selected by the Heureka system for the productive Swedish forest land (represented by NFI plots), given combinations of Climate Change Mitigation Solutions and climate scenarios. The percentage of area is given by the frequency of application of the different management regimes among NFI plots.


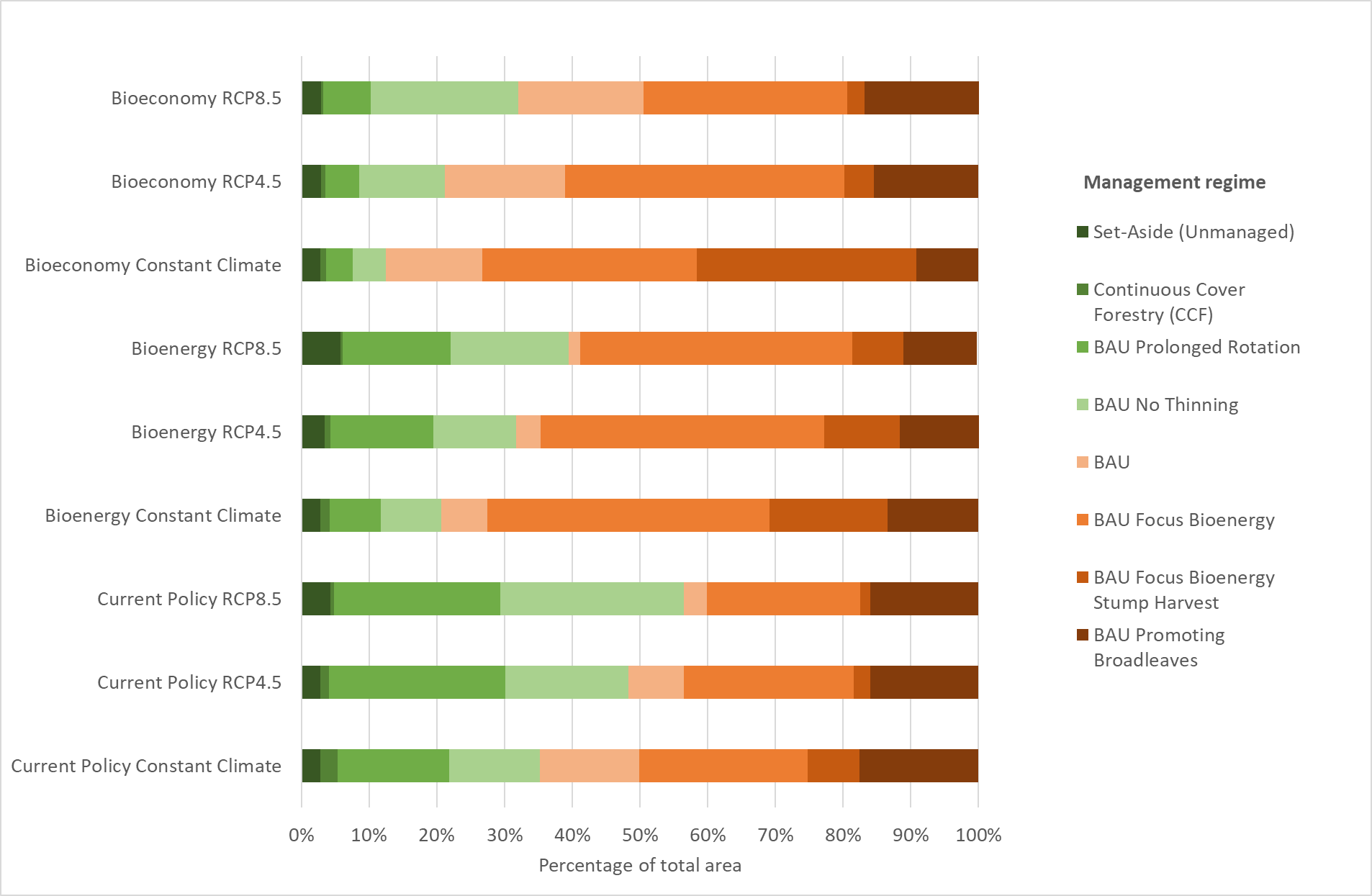


1. **Building the climate scenarios**

The CCMS scenarios and forest dynamics and management were projected assuming three climate scenarios. The first one was Constant Climate, specifically averages of the climate predictors for the period 1983-1992 (the period on which the Heureka growth models are based, Fahlvik et al., 2014) maintained constant for the whole projection period. The other two scenarios were two IPCC radiative forcing scenarios, Representative Concentration Pathways (RCPs, van Vuuren et al., 2011), RCP4.5 and RCP8.5. Concerning forest projections, Heureka allows specifying using either of these scenarios in the software where the forest growth is affected. This further affects tree biomass, the output ES from Heureka (net biomass accumulation, carbon storage in trees and soil, deadwood volume) and the forest predictors of the models for the non-wood ES stand age and biomass of the main tree species (spruce, pine, and birch) and also the timing of management regimes. As climate predictors for the non-wood ES, we extracted temperature and precipitation sums and Psum for the projection period for the coordinates of the NFI plots, following the procedure in Mair et al. (2018).

Climate projections based on RCP 4.5 assume moderate GHG emission reduction (range 2-4.5 °C by 2100 for Sweden), and the RCP 8.5 assumes no emission mitigation undertaken (range 4-7 °C for Sweden). The Swedish Meteorological and Hydrological Institute (SMHI) has developed a high-resolution database (EURO4M) of surface meteorological variables (5 x 5 km^2^) covering the period 1989-2010 from which it is possible to calculate climate indices useful for prediction as in the current study. Both the RCP scenarios were ensembled across five different global climate models (CNRM-CM5, EC-EARTH, HADGEM2-ES, IPSL-CM5A-MR, MPI-ESM-LR) from the CMIP5 archive (Taylor et al., 2012). To increase the spatial resolution in the climate scenarios, data from the global models were used to force the regional climate model RCA4 (Stralberg et al., 2015). To further increase the resolution and reduce model biases, the results were bias adjusted using a distribution-based scaling (DBS) method (Yang et al., 2010a, b).

**REFERENCES**

Capros, P., De Vita, A., Hoeglund-Isaksson, L., Winiwarter, W., Purohit, P., Bottcher, H., Frank, S., Havlik, P., Gusti, M., & Witzke, H.P. (2013). *EU energy, transport and GHG emissions trends to 2050 reference scenario.* European Commission, Luxembourg.

Fahlvik, N., Elfving, B., & Wikström, P. (2014). Evaluation of growth models used in the Swedish Forest Planning System Heureka. *Silva Fennica*, *48*(2), 1013. https://doi.org/10.14214/sf.1013

Fältinstruktion. (2021). *Instruktion för fältarbere vid Riksinventeringen av skog [Fieldwork instructions for the Swedish National Forest Inventory]*. Umeå: Department of Forest Resource Management and Geomatics, Swedish University of Agricultural Sciences . In English. https://www.slu.se/globalassets/ew/org/centrb/rt/dokument/faltinst/nfi_fieldwork_instructions_eng.pdf

Forsell, N., Korosuo, A., Havlík, P., Valin, H., Lauri, P., Gusti, M., Kindermann, G., Obersteiner, M., Böttcher, H., Hennenberg, K., Hünecke, K., Wiegmann, K., Pekkanen, M., Nuolivirta, P., Bowyer, C., Nanni, S., Allen, B., Poláková, J., Fitzgerald, J., & Lindner, M. (2016). *Study on impacts on resource efficiency of future EU demand for bioenergy (ReceBio). Final report.* http://ec.europa.eu/environment/enveco/studies.htm#4

Gamfeldt, L., Snäll, T., Bagchi, R., Jonsson, M., Gustafsson, L., Kjellander, P., ... & Mikusiński, G. (2013). Higher levels of multiple ecosystem services are found in forests with more tree species. *Nature communications*, 4, 1340.

Gelman, A., Carlin, J.B., Stern, H.S. & Rubin, D.B. (2004). *Bayesian Data Analysis 2nd edn*. CRC Press.

Gelman, A., Goodrich, B., Gabry, J., & Vehtari, A. (2019). R-squared for Bayesian regression models. *The American Statistician*, *73*(3)*,* 307-309.

Gilks, W. R., Thomas, A., & Spiegelhalter, D. J. (1993). Software for the Gibbs sampler. *Computing Science and Statistics*, 439-439.

Goudie, R. J., Turner, R. M., De Angelis, D., & Thomas, A. (2020). MultiBUGS: A parallel implementation of the BUGS modelling framework for faster Bayesian inference. *Journal of statistical software*, *95*.

Havlik, P., Valin, H., Schmid, E., Herrero, M., Mosnier, A., & Obersteiner, M. (2015). Climate change impacts and mitigation in the developing world: an integrated assessment of the agriculture and forestry sectors. *World Bank Policy Research Working Paper, 7477*. https://doi.org/10.1596/1813-9450-7477

Mair, L., Jönsson, M., Räty, M., Bärring, L., Strandberg, G., Lämås, T., & Snäll, T. (2018). Land use changes could modify future negative effects of climate change on old‐growth forest indicator species. *Diversity and Distributions*, *24*(10), 1416-1425.

Stralberg, D., Bayne, E. M., Cumming, S. G., Sólymos, P., Song, S. J., & Schmiegelow, F. K. A. (2015). Conservation of future boreal forest bird communities considering lags in vegetation response to climate change: a modified refugia approach. *Diversity and Distributions, 21*(9), 1112–1128.

Spiegelhalter, D. J., Best, N. G., Carlin, B. P., & van der Linde, A. (2002). Bayesian measures of model complexity and fit. *Journal of the Royal Statistical Society: Series B (Statistical Methodology)*, *64*(4), 583–639. https://doi.org/10.1111/1467-9868.00353

Taylor, K. E., Stouffer, R. J., & Meehl, G. A. (2012). An overview of CMIP5 and the experiment design. *Bulletin of the American Meteorological Society*, *93*(4), 485–498. https://doi.org/10.1175/BAMS-D-11-00094.1

van Vuuren, D. P., Edmonds, J., Kainuma, M., Riahi, K., Thomson, A., Hibbard, K., ... & Rose, S. K. (2011). The representative concentration pathways: an overview. *Climatic Change*, *109*(1), 5-31.

Yang, W., Andréasson, J., Graham, L. P., Olsson, J., Rosberg, J., & Wetterhall, F. (2010a). Distribution-based scaling to improve usability of regional climate model projections for hydrological climate change impacts studies. *Hydrology Research*, *41*(3–4), 211–229. https://doi.org/10.2166/nh.2010.004

Yang, W., Bárdossy, A., & Caspary, H.J. (2010b). Downscaling daily precipitation time series using a combined circulation- and regression-based approach. *Theoretical and Applied Climatology*, *102*(3), 439–454. https://doi.org/10.1007/s00704-010-0272-0

**Appendix 3. GLMs for evaluation of the effects of climate and CCMS scenarios on ES**

We applied frequentist GLMs to evaluate separately the effect of climate and CCMS scenarios and their interactions on levels of ES and ES multifunctionality summarizing their values at the beginning (2020), in the middle (2060) and at the end (2100) of the considered projection horizon separately in production forests and in the *Set-aside* scenario (Tables S3-S4).

When fitting GLMs to the output of the projections, mean estimated effects for the NFI plots were reported along with standard errors. *t* tests and relative *p* values were used to address the magnitude of the effect of each scenario and its *p* value, under the null hypothesis of no effect. Significance was set at p < 0.05. In the GLMs, we assumed a normal distribution and used an identity link function for net biomass accumulation, deadwood volume, tree C storage, soil C storage and ES multifunctionality (all log-transformed). To account for the non-normal distribution of bilberry cover and wildfood plant cover, we assumed for both a normal distribution and an identity link function but with an arcsine transformation for the first and with a log transformation for the second. For the counts of understory richness, we assumed an over-dispersed Poisson distribution and a log link function.

**Table S3**. Generalized Linear Models (GLMs) summarizing the effects of **Climate Change Mitigation Solutions scenarios** (Current Policy, Bioenergy, and Bioeconomy) projected on production land, climate scenarios (Constant Climate, RCP4.5, and RCP8.5) and their interaction on future levels of ES and on Multifunctionality every 40 years over the 21^st^ century. The Intercept refers to the mean and standard error in transformed level of the focal ES given the scenarios Current Policy and Constant Climate. The remaining Estimates and test statistics (*t*- and *p*-values) concern difference in (transformed) ES levels compared to this scenario. The null hypothesis is no difference compared to this base scenario. Pr(>|t|) equal to 0 represent values lower than 0.001.

**Table S4**. Generalized Linear Models (GLMs) summarizing the effects of climate scenarios (Constant Climate, RCP4.5, and RCP8.5) on future levels of ES and on Multifunctionality every 40 years over the 21^st^ century **in the *Set-aside* scenario** (not varying among policy scenarios). The Intercept refers to the mean and standard error in transformed level of the focal ES given the scenarios Constant Climate. The remaining Estimates and test statistics (*t*- and *p*-values) concern difference in (transformed) ES levels compared to this scenario. The null hypothesis is no difference compared to this climate scenario. Pr(>|t|) equal to 0 represent values lower than 0.001.
